# A CNN-Based Image Detection System for Full-Surface Defects in Brown Rice

**DOI:** 10.3390/foods15162910

**Published:** 2026-08-20

**Authors:** Zhaoyan You, Jianchun Yan, Hai Wei, Minji Liu, Jiannan Wang, Huanxiong Xie

**Affiliations:** 1 Nanjing Institute of Agricultural Mechanization, Ministry of Agriculture and Rural Affairs, Nanjing 210014, China; 2 College of Engineering, Nanjing Agricultural University, Nanjing 210031, China

**Keywords:** image recognition, convolutional neural networks, Yolov5s, brown rice, full-surface defects, smart food inspection

## Abstract

In order to enable accurate and efficient rice quality evaluation through full-surface defect detection of brown rice, a detection system based on convolutional neural network (CNN) was developed. A dataset of images of five categories—unhulled, normal, broken, cracked, and insect-bitten brown rice—was collected. Three CNN models, YOLOv5s, YOLOv7, and Faster R-CNN, were evaluated and compared with traditional algorithms including support vector machine (SVM) and back propagation (BP) neural networks. Experimental results showed that CNN-based methods in the present database significantly outperformed traditional approaches, with the YOLOv5s model achieving the best comprehensive performance: 95.80% detection accuracy, 10.90 ms inference time per image, and 92 frames/s processing speed. An improved Rice-YOLOv5s algorithm was further proposed and validated through batch detection experiments, achieving an average recognition accuracy of 96.44% and a processing time of 9.2 ms per image, which is equivalent to approximately 108.7 FPS. This study demonstrates the feasibility of CNN-based brown rice defect detection, with future work directed toward lightweight deployment and multimodal fusion for production-line application.

## 1. Introduction

According to statistics from the Food and Agriculture Organization of the United Nations, the global rice production in 2026 is estimated at 561.6 million tons, and about 20% of the world’s food calories are provided by rice. More than half of the 8.02 billion people on earth heavily rely on rice, and it is expected that by 2030, global demand for rice will increase by 25% [1,2,3,4]. Rice is prone to varying degrees of quality damage in various stages such as harvesting, transportation, drying, and storage [5,6,7], for example, phenomena such as breakage, cracking, insect-bitten, and mildew transformation may occur [8,9,10]. Efficient, non-destructive, and rapid rice quality testing technology plays an important role in promoting the healthy development of the rice industry [11,12]. Currently, many crops can be quickly and accurately quality-assessed through full-surface defect detection [13,14]; rice selection can also be evaluated based on appearance quality indicators using the mechanical force generated by the rice milling machine to remove rice husks [15]. Therefore, the automatic surface defect identification system of brown rice can be developed to effectively improve the level of rice quality intelligent detection and identification efficiency.

Traditional rice quality inspection is mainly performed by manual visual judgment, which is inefficient, subjective, and labor-intensive, and thus cannot meet the requirements of modern grain processing for real-time, high-throughput, and consistent testing. Thanks to the rapid development of computer vision and deep learning, machine vision systems are now being adopted in agricultural quality inspection, offering new ways to achieve automated and intelligent defect detection. Nowadays, many researchers have conducted research on the identification of different rice varieties, batches, and appearance quality indicators using visual image recognition methods, optical non-destructive testing techniques, or predictive analysis models. An innovative rice contact segmentation technique combined with damage estimation based on geometric characteristics and crack estimation using new image analysis was used to evaluate damage and cracks of steamed rice grains [16]. On image analysis, visible and near-infrared spectroscopy was used to determine the three-dimensional size and crack changes in rice during water absorption [17]. A visible light near-infrared hyperspectral imaging (HSI) system with a xenon light source was used for the hypercube data visualization of the fresh and insect-infested rice grains to obtain the first and second principal component maps of insect-infested and healthy rice grains [18]. A novel non-destructive analysis method for the purity of multi-grain rice seeds [19] was developed by using visible and near-infrared spectroscopy. A rice quality analysis device based on visual recognition technology was designed to establish a Canny edge detection algorithm model by using MatLab R2018a software, achieving detection of the number of rice grains and recognition of cracked rice grains after drying [20]. RGB and hyperspectral imaging analysis techniques were investigated to discriminate the varieties and qualities of rice seeds [21,22]. Experiments and numerical simulations were performed in an attempt to predict the cracking behavior of germinated brown rice under different drying stages through intragranular drying kinetics [23]. An improved two-stream convolutional neural network model was proposed based on ECA to classify rice by single grain weight [24]. Research on automatic detection of yellow grain rice or whole polished rice based on image recognition has also been conducted [25,26]. A prediction classification model based on an IFA-SVM (improved firefly algorithm-support vector machine) was proposed to determine whether mildew occurred in rice under the given temperature, moisture, and storage time [27].

The convolutional neural network is one of the representative algorithms of deep learning and can automatically learn feature representation of images [28,29,30]. There are many types of CNN models, such as VGG [31,32], ResNet [33], LeNet [34], MobileNet [35], R-CNN series [36], YOLO series [37,38,39,40,41,42], SSD [43], etc. In the field of agriculture, CNN models can achieve end-to-end detection by learning target features from different fields, scenes, and scales, and have become a research hotspot in agricultural information technology [44,45]. There are image detection systems for wheat grain integrity and corn seed internal cracks based on CNN [46,47]. A segmentation method for grains, branches, and stems in harvested rice grain images was proposed based on the U-Net deep learning model [48]. The transfer learning mechanism has been introduced and combined with improved CNN models to design detection and recognition systems for grape leaf diseases, corn diseases, and other complex-background scenarios [49,50]. An improved algorithm based on CNN and SVM classification optimization was developed for automatic recognition and correction of garlic bulb orientation [51]. County-level wheat yield prediction based on CNN and BP neural networks has also been reported [52].

Despite the progress reviewed above, three critical gaps remain: single-surface imaging cannot detect defects on the dorsal side; handcrafted features are sensitive to lighting variations; and deep-learning approaches have rarely been explored in this domain. To address these gaps, we systematically compare traditional algorithms (SVM, BP) with several CNN models (YOLOv5s, Faster R-CNN, YOLOv7) to select the optimal model, thereby providing technical support for the visual recognition of full-surface defects in brown rice. We have restated our study objectives as explicit research questions, where the two research questions are: (1) Can the proposed model achieve significantly higher detection accuracy than conventional methods (SVM, BP) and the baseline YOLOv5s for full-surface brown rice defect detection? (2) How much do the CA attention mechanism, BiFPN, and the small-object detection layer individually contribute to the overall performance improvement?

This study falls within the emerging field of intelligent grain quality monitoring, where computer vision and deep learning are increasingly being adopted as non-destructive analytical tools for automated grain inspection. Specifically, our proposed system mimics human visual assessment while offering superior speed, consistency, and objectivity. By integrating image acquisition, CNN-based defect detection, and real-time decision-making, this work contributes to the development of smart analytical technologies for grain quality control.

## 2. Materials and Methods

### 2.1. Brown Rice Full-Surface Defect Recognition Test-Bed

**Figure 1:**
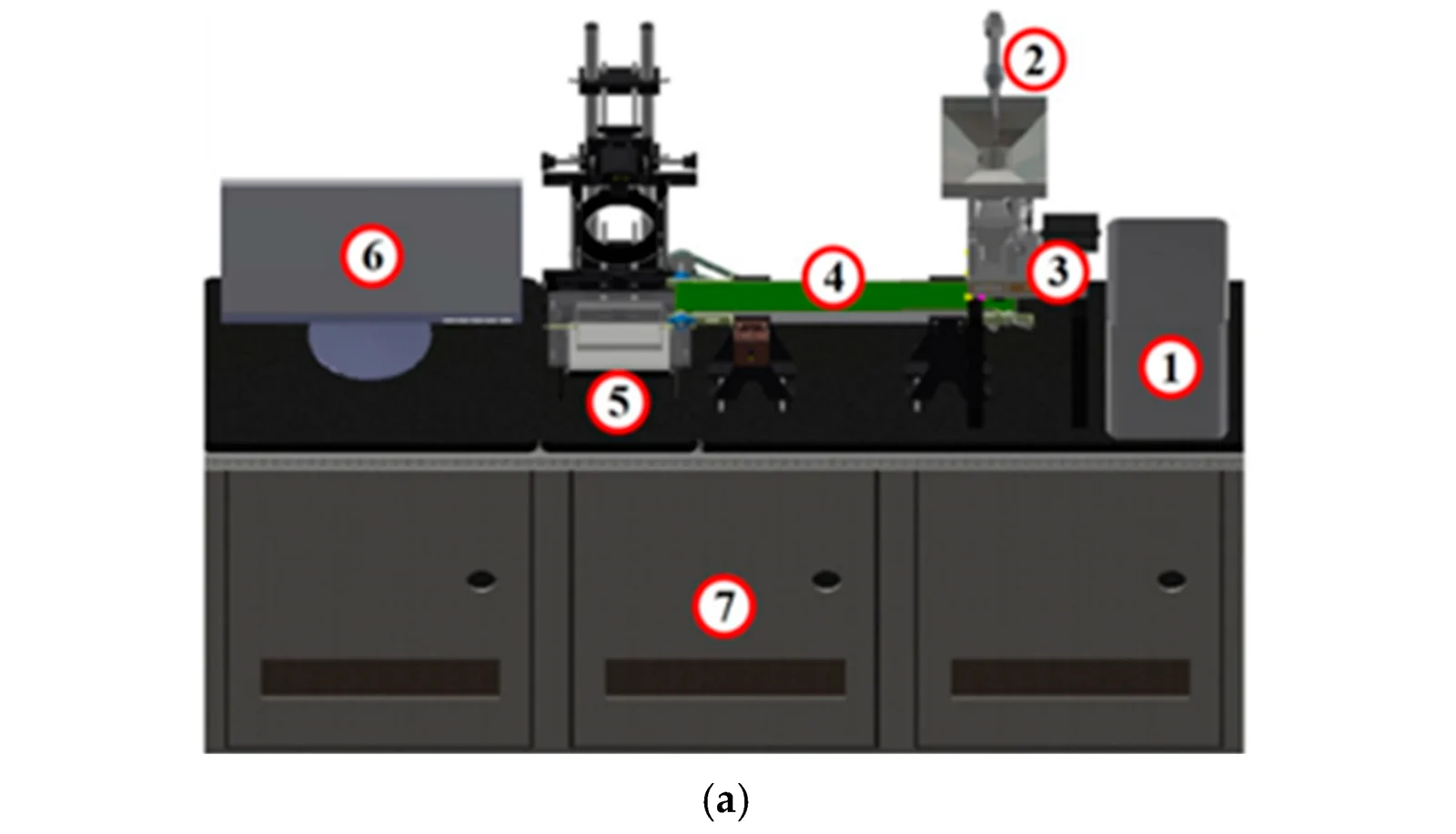

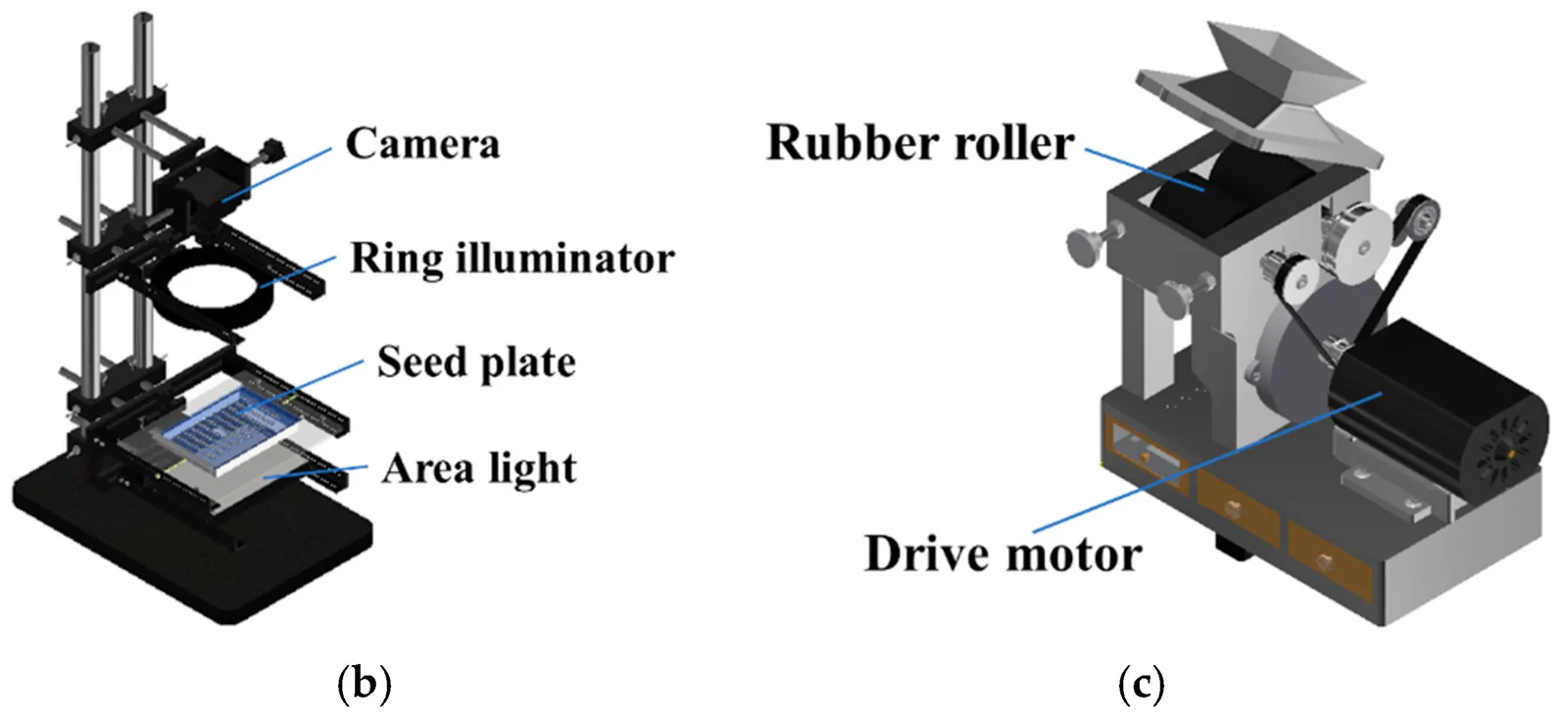
Overview of the brown rice full-surface defect recognition test-bed and its key components. (**a**) Brown rice full-surface defect recognition test-bed. Main components: 1. Material box 2. Rice anti-blocking module 3. Rice husker 4. Conveying module 5. Optical image acquisition module 6. Upper computer 7. Control box. (**b**) Image acquisition platform. (**c**) Rice husker.

The developed brown rice full-surface defect recognition test-bed is shown in Figure 1, which mainly includes a material box, an anti-blocking module, a husking unit, a conveying module, an optical image acquisition module, an upper computer, and a control box. The material box is used to store the rice to be inspected. The anti-blocking module ensures smooth feeding of rice into the husker from the funnel. The driving motor on the rice husker drives two rubber rollers to move in opposite directions, thereby removing the husks on the surface of the rice that fall between the rubber rollers. By adjusting the rotating nut above the rice husker, the gap between the rubber rollers can be easily adjusted, ensuring that complete brown rice is obtained without creating additional broken rice. The conveying unit is a conveyor belt driven by a stepper motor equipped with a speed regulator, responsible for transferring the brown rice from the husking unit to the image acquisition unit. The image acquisition unit can clearly capture the full-surface defects of the husked brown rice, and the recognition system in the upper computer can identify full-surface defects such as unhulled, cracked, broken, and insect-bitten. The control circuit module handles the power supply and circuit control of the above work units. Therefore, the designed experimental platform can achieve integrated functions including brown rice sampling, milling, conveying, and intelligent defect recognition in a single pass.

### 2.2. Image Acquisition and Dataset Construction

Rice is the largest grain crop in Jiangsu Province, with a planting area of 33.47 million mu in 2025. This article selects the representative rice variety Nanjing 46, a japonica conventional rice, bred by the Grain Crop Research Institute of Jiangsu Academy of Agricultural Sciences. This rice variety has strong regional representativeness and a wide planting area in Jiangsu. Rice samples were collected from a field in October 2025, located at 31°17′ N and 120°01′ E.

#### 2.2.1. Multiple Defect Categories

**Figure 2:**
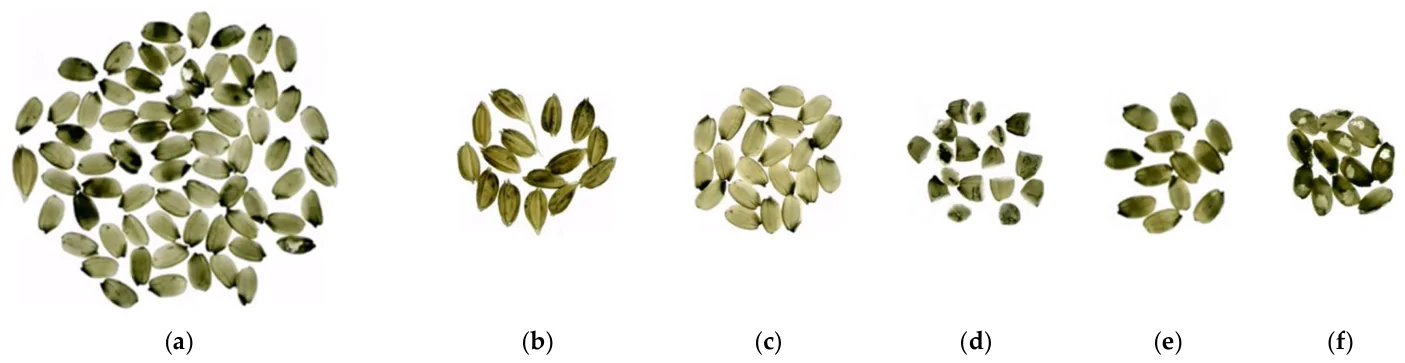
Common sample classification of brown rice. Note: (**a**) unsorted brown rice, (**b**) unhulled rice, (**c**) normal brown rice, (**d**) broken brown rice, (**e**) cracked brown rice, (**f**) insect-bitten brown rice.

The surface defects of brown rice can be systematically divided into five categories based on shape, color, texture, integrity, perimeter, and other characteristics, as shown in Figure 2, which are unhulled, normal, broken, cracked, and insect-bitten. Rice with surface defects is often caused by harvest damage or improper drying or storage, which can lead to reductions in germination rate and quality of rice, affecting rice processing and storage and thereby reducing its market value. Specific descriptions of these five categories of brown rice are as follows:

(1) Unhulled rice: Brown rice with rice husk not removed due to excessive spacing between milling rubber rollers.
(2) Normal brown rice: Brown rice with a relatively hard shell texture, a smooth and glossy surface and brown or golden in color.
(3) Broken brown rice: Brown rice with damage exceeding one-fifth of the grain volume.
(4) Cracked brown rice: Brown rice with cracks in the embryo, or a clear transverse crack running through the entire grain, or two or more transverse cracks, or longitudinal cracks exceeding two-thirds of the grain length.
(5) Insect-bitten brown rice: Brown rice eroded by insects, with damage to the embryo or endosperm.

#### 2.2.2. Selection of Light Source for Full-Surface Defect Identification

**Figure 3:**
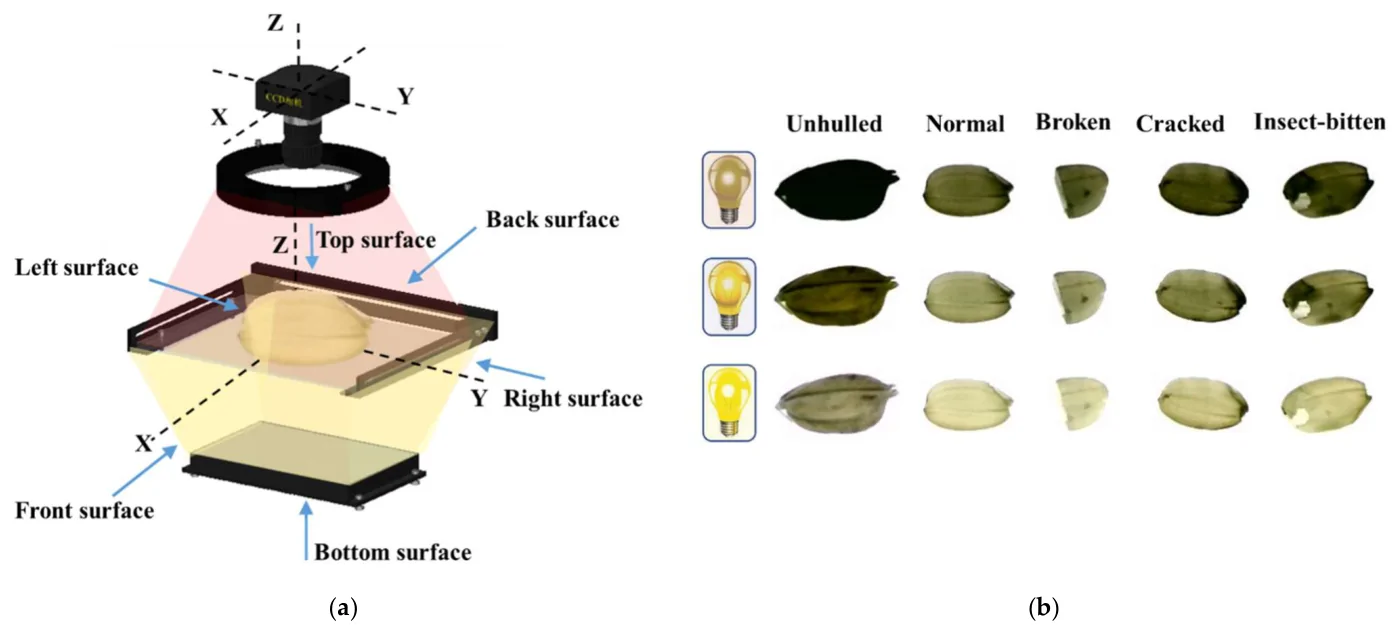
Brown rice image acquisition system and defect visualization comparison under varying illumination. (**a**) Schematic diagram of the image acquisition under the light source combination. (**b**) Brown rice defect visualization comparison under varying illuminations.

The hulled rice grains are randomly dropped onto a transparent seed placement plate through the conveying device. As the seeds randomly fall onto the seed placement plate, when a single light source is used from top to bottom or from bottom to top, the internal cracks of seeds on the light source side can be clearly imaged. However, defects on the opposite side or cracks within the kernel may be missed or not recognized. The schematic diagram of the image acquisition light source combination is shown in Figure 3a, a combination of upper and lower light sources is needed to visualize full-surface defects of brown rice. In the process of image acquisition process, light sources were installed above and below the sample plate. The upper circular light source model is 150–90 W, with voltage of 24 V, power of 14 W, and color temperature 6000–6500 K, providing illumination from a 360-degree view to cover the entire sampled brown rice and detect defects or surface features at different angles. The lower panel light source model is 100 W, with voltage of 24 V, power of 22 W and color temperature 6000–6500 K, providing good lighting with relatively uniform illumination for the area. This combination of light sources can reveal defects or surface features of the entire sampled brown rice. Different brightness environments can expose different texture features in brown rice during image acquisition. Therefore, image acquisition with multiple brightness environments can help neural networks learn more defect features and determine the most suitable brightness. In this study, brightness was adjusted using a two-channel visual light source controller, model JS-AC24V100W-2T, manufactured by Shenzhen Optical Instrument Factory (Shenzhen, China), with low brightness at 6000–6150 K, medium brightness at 6200–6300 K, and high brightness at 6350–6500 K. Figure 3b shows a visualization comparison of brown rice defects under different illumination conditions: it can be seen that when the brightness of the light source is moderate, the light can fully transmit through and illuminate the entire surface defects of brown rice from both sides.

#### 2.2.3. Dataset Processing

The rice variety used in this study was Nanjing 46, bred by the Institute of Food Crops, Jiangsu Academy of Agricultural Sciences. Images were acquired using a GI4300-MT9F002 industrial digital color camera, manufactured by Shenzhen Optical Instrument Factory, equipped with an Aptina MT9F002 CMOS image sensor, which was originally launched by the American company Aptina (San Jose, CA, USA, 14 effective megapixels) and an 8 mm fixed-focus lens. The exposure time was set to 20 ms. Illumination was provided by a combined lighting system consisting of an upper ring light (model 150–90 W, power 14 W, color temperature 6000–6500 K) and a lower panel light (model 100 W, power 22 W, color temperature 6000–6500 K). The brightness was regulated using a two-channel visual light controller, and a medium brightness setting (color temperature 6200–6300 K) was adopted. During image acquisition, the working distance between the camera and the sample plate was maintained at 125–200 mm, and the camera was positioned perpendicular (90°) to the horizontal plane. All images were uniformly resized to 640 × 640 pixels and saved in .jpg format.

**Figure 4:**
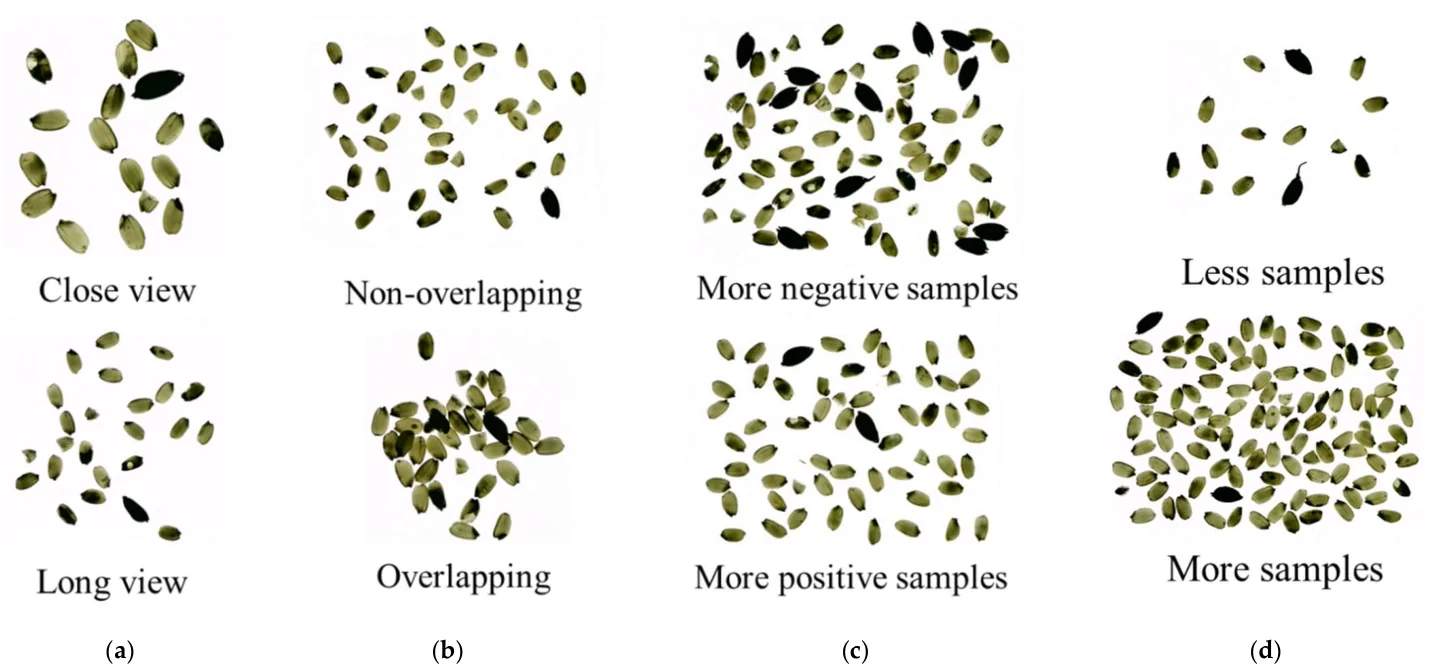
Image acquisition of brown rice samples in different scenarios. (**a**) Close view and long view. (**b**) Non-overlapping and overlapping. (**c**) Different positive and negative samples. (**d**) Number of samples in a single image.

Figure 4 shows the image acquisition of brown rice samples under different scenarios. Data collection was carried out from September to December 2025, and a total of 1080 original images were collected, including close views and long views, varying degrees of overlap, different positive and negative samples, and varying numbers of targets per image. The rectangular annotation tool LabelImg was used to manually label the collected images for defect categories and location information. The dataset was saved in YOLO format, thus completing the construction of the brown rice dataset. The dataset was strictly divided by batch, rather than by individual image. Each acquisition batch consisted of approximately 5 to 120 brown rice grains, and all batches were collected independently. In this study, the 10 acquisition batches were split in a 7:2:1 ratio, with 7 batches allocated to the training set (756 images), 2 batches to the validation set (216 images), and 1 batch to the test set (108 images). This splitting strategy ensured that no images from the same batch (or batches containing similar samples) appeared simultaneously across the training, validation, or test sets, thereby effectively avoiding data leakage.

For the batch detection experiments, 500 brown rice kernels (100 per category: unhulled, normal, broken, cracked, and insect-damaged) were randomly selected from the independent validation set (200 images) using a random number generator to ensure representativeness. The selection was performed separately for each category to maintain a balanced class distribution. The 500 kernels were then thoroughly mixed into a single group and randomly subdivided into five equal parts (100 kernels each). For each part, one image was captured under the same illumination and camera settings. This process was repeated for 10 independent test rounds, with the same random sampling and subdivision procedure applied consistently across all rounds to ensure reproducibility.

## 3. Identifying Algorithm and Improvement for Brown Rice Full-Surface Defects

### 3.1. Model Selection and Analysis

For the single-stage object detection algorithm, YOLOv5s has a relatively small network depth and feature map width, providing faster inference speed, and is widely used in practical scenarios. Therefore, YOLOv5s is used as the basic model for this study, and its network structure includes four parts: input, backbone, neck, and prediction. The input side adopts the mosaic data augmentation method, randomly scaling, cropping, and arranging several images before concatenating them to enrich the dataset and enhance model generalization. By calculating the optimal anchor box parameters through adaptive anchor boxes, the accuracy and robustness of the model are improved. The backbone network combines focus slicing and spatial pyramid pooling fast (SPPF) to extract features. The neck section adopts a combination of feature pyramid networks (FPN) and path aggregation networks (PANs). FPN conveys semantic information from top to bottom, while PAN integrates positional information from bottom to top, thereby obtaining richer feature information. The prediction section contains three prediction branches, which use the extracted feature information to predict targets of different sizes and obtain the category, confidence, and position information of the predicted targets. Meanwhile, YOLOv7 and Faster R-CNN, two other classic convolutional neural network models in deep learning, were selected for comparative analysis to explore the optimal model suitable for detecting surface defects of brown rice.

Another 200 brown rice images from an independent validation set (newly collected after training, used only for final performance comparison) were collected from the experimental materials as test data, and the weight files of Faster R-CNN, YOLOv5s, and YOLOv7 were loaded and run for 500 iterations for testing. The experimental results are shown in Table 1. YOLOv5s has a shorter inference time per image than Faster R-CNN and YOLOv7, and YOLOv5s has higher recognition accuracy for brown rice surface defects, reaching 95.80%. The average inference detection time per image is 10.90 ms. To compare the accuracy of CNN algorithms and traditional algorithms in identifying brown rice surface defects, two traditional algorithms with good generalization ability, SVM (support vector machine) and BP (back propagation) neural network were selected to test the same samples. As for SVM, we used a radial basis function (RBF) kernel with hyperparameters optimized via grid search (C = 10, γ = 0.01). The model was trained as a one-vs-rest multi-class classifier. As for BP, we implemented a three-layered fully connected network with 64 hidden neurons, ReLU activation, and a Softmax output layer. The model was trained using the Adam optimizer with a learning rate of 10^−5^ and a batch size of 20 for 500 epochs. Both models were trained on the same training set (756 images) and evaluated based on the same test set (108 images) as the CNN models. For each image, the feature vector was averaged across all detected kernels to obtain a single sample per image.

The results are shown in Table 1, in terms of recognition accuracy and detection speed; both traditional algorithm models were inferior to the convolutional neural network models.

### 3.2. YOLOv5s Model Improvement

**Figure 5:**
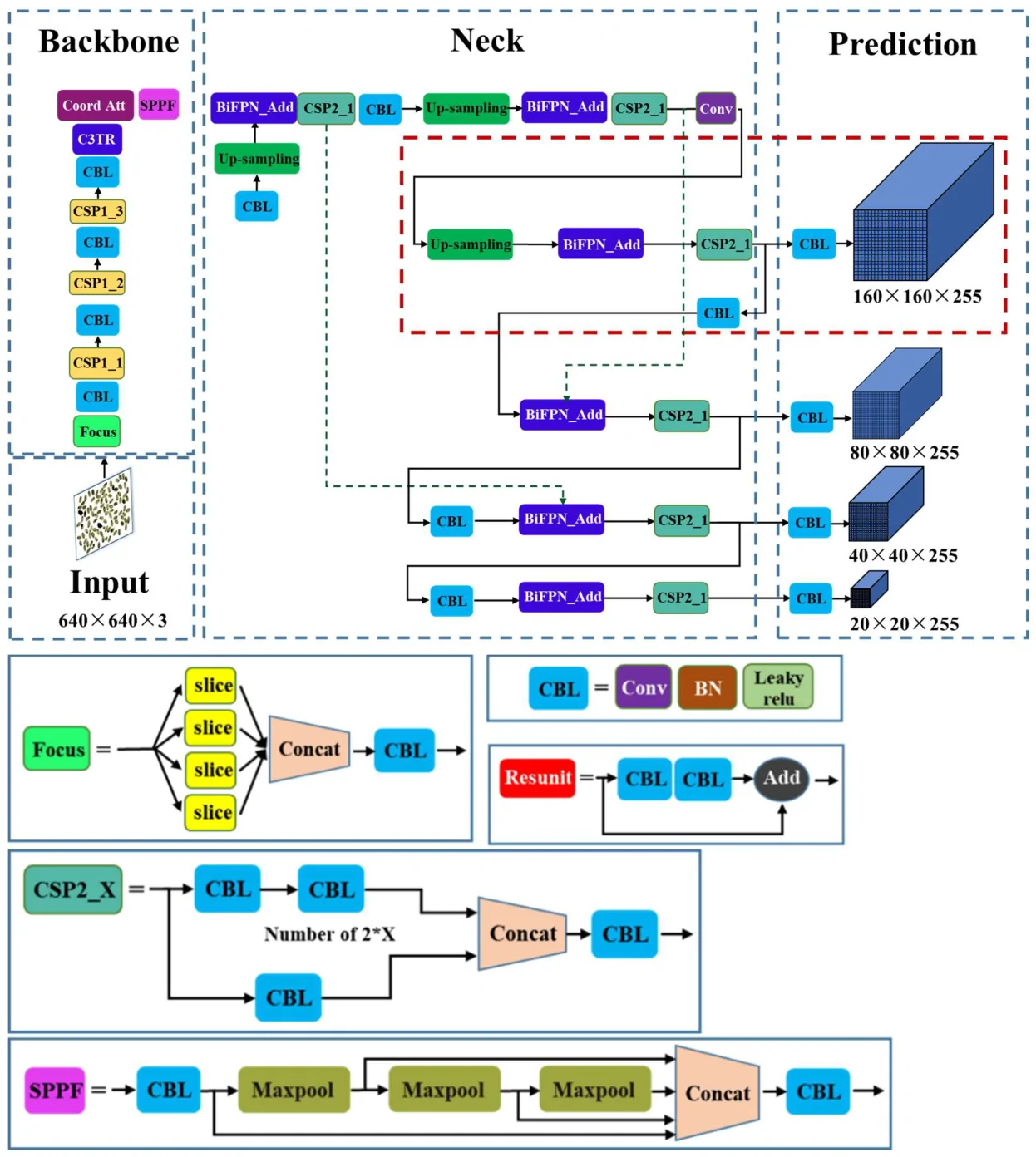
Rice-YOLOv5s model structure diagram. Abbreviations: conv, convolution; BN, batch normalization; leaky relu, activation function; concat, tensor splicing; add, tensor addition; resunit, residual module; maxpool, maximum pooling operation; CBL module is composed of conv, BN layer and leaky relu activation function; CSP1_X is a convolution structure integrated by X residual components; CSP2_X is a convolution structure integrated by 2 × X CBL modules; SPPF is a spatial pyramid pooling structure.

Under experimental conditions and practical applications, the brown rice surface defects dataset constructed in this study has a relatively single scene, with only brown rice as the detection target and a non-complex background. A model with fewer parameters can fit the brown rice dataset. However, due to the presence of small target defects such as minor insect damage or fine cracks on the surface or inside of brown rice, overlapping defects may also occur during partial stacking of brown rice, which can easily lead to false or missed detections. Therefore, it is necessary to improve the backbone, neck, and detection parts of the YOLOv5s model and construct the Rice-YOLOv5s model structure, as shown in Figure 5. The Rice-YOLOv5s model is based on the YOLOv5s network for extracting multi-scale features. The improved network is optimized by an anchor box generation mechanism at the input stage. Firstly, the C3 module in the backbone part is replaced by a C3TR with a transformer. Then, a coordinate attention mechanism is added after the C3TR module. In the neck stage, a more efficient weighted bidirectional feature pyramid network (BiFPN) structure is used as the neck, and a small object detection layer is added in the detection section to prevent minor defects from being missed.

#### 3.2.1. CA Attention Mechanism

**Figure 6:**
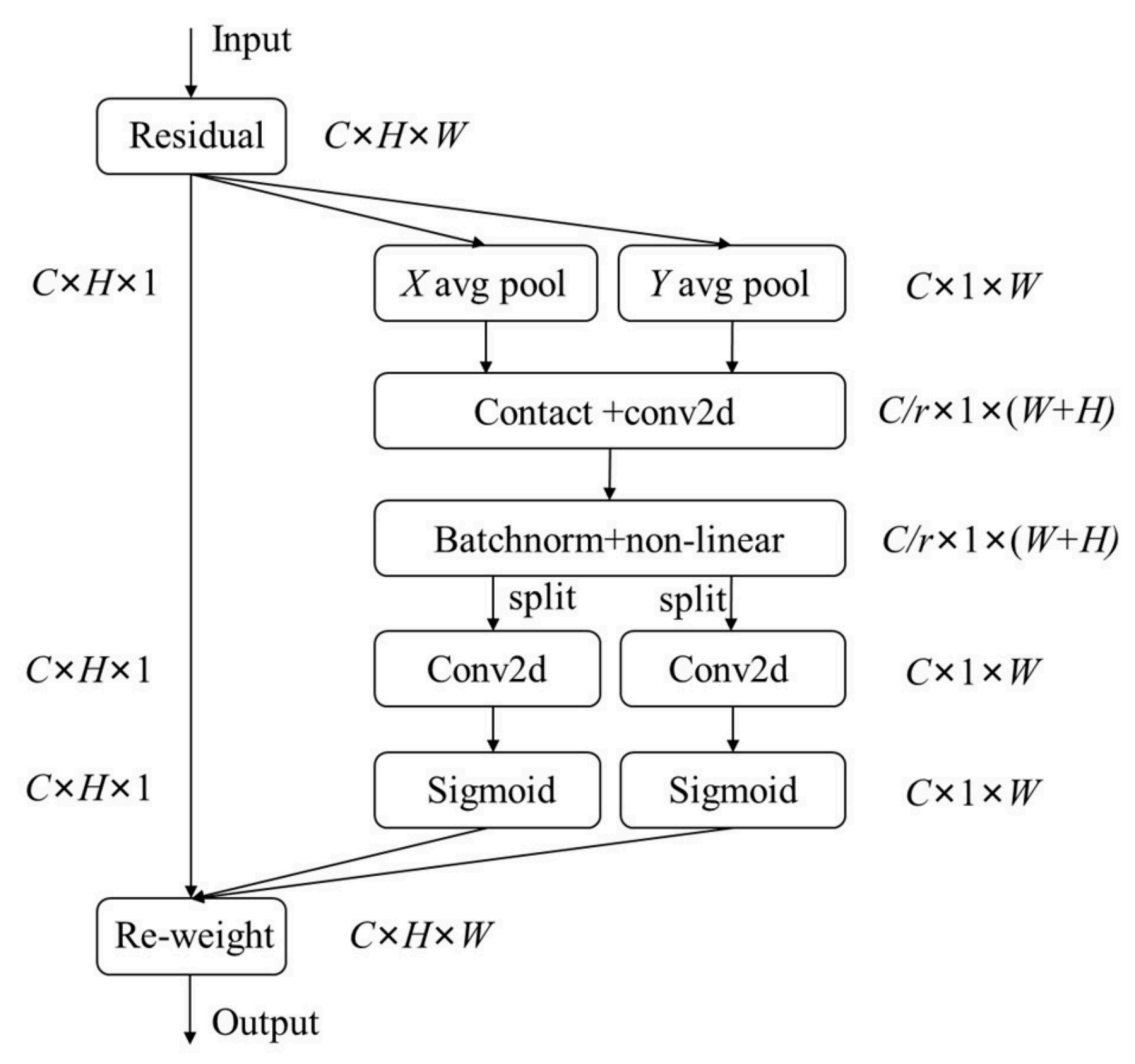
Structure diagram of coordinate attention mechanism. Note: *C*, *H*, and *W* denote the number of channels, height, and width of the input feature map, respectively, and *r* denotes the channel downsampling rate.

An attention mechanism enables deep learning networks to pay more attention to the regions that need attention, which is of great help in improving the performance of neural networks [53]. Currently, it is widely used in computer vision tasks, but conventional attention mechanisms are prone to ignoring target spatial position information. The CA (coordinated attention) attention mechanism fully considers the correlation between channel information and directional position information, helping the model better locate and recognize defect features [54]. The CA structure is shown in Figure 6, and the specific implementation process is as follows:

(1) Firstly, given the input layer *X* (size *C* × *H* × *W*), pooling kernels of size (*H*, 1) and (1, *W*) were used to encode each channel horizontally and vertically, respectively. The outputs of the *c*-th channel with height *H* and the *c*-th channel with width *W* are shown in Equations (1) and (2):

(1) $$S_{c}^{h}(h)=\frac{1}{W}\sum_{0\leq i\leq w}x_{c}(h,i)$$

(2) $$S_{c}^{w}(w)=\frac{1}{H}\sum_{0\leq j\leq H}x_{c}(j,w)$$

In the formula, *H* and *W* respectively represent the height and width of the feature layer; $S_{c}^{h}(h)$ and $S_{c}^{w}(w)$ represent the outputs of the *c*-th channel along the *H* and *W* directions; $x_{c}(h,i)$ and $x_{c}(j,w)$ represent the inputs of feature map *X* along the *H* and *W* directions.

(2) Coordinate attention generation is performed by the concatenation, 1 × 1 convolution, and activation of abovementioned feature maps from the two independent directions, as shown in Equation (3).

(3) $$e=s\left\{C_{1}([S^{h},S^{w}])\right\}$$

In the formula, *e* represents the intermediate feature map after encoding spatial information in the horizontal and vertical directions; [$S^{h},S^{w}$] represents the concatenation operation along the spatial dimension; *C*_1_ represents the convolution operation, and *s* represents the Sigmoid function. Then, *e* is split along the spatial dimension, and two 1 × 1 convolution operations are used to restore the number of channels in the feature map to the same number as the input feature map *X*. Finally, the attention weight of the feature map is calculated using the sigmoid function, and the results are shown in Equations (4) and (5): in the formula, *g^h^* and *g^w^* represent the attention weights for the convolution and activation after *e* splitting; *e^h^* and *e^w^* represent the feature maps along the *H* and *W* directions after *e* splitting, respectively; *C_h_* and *C_w_* represent the convolution operation.

(4) $$g^{h}=\tau (C_{h}(e^{h}))$$

(5) $$g^{w}=\tau (C_{w}(e^{w}))$$

(3) Multiply and weigh *g^h^*, *g^w^* with the input feature map weights to output a feature map with coordinate attention weights, as shown in Equation (6).

(6) $$y_{c}(i,j)=x_{c}(i,j)\cdot g_{c}^{h}(i)\cdot g_{c}^{w}(j)$$

In the equation, $y_{c}(i,j)$ and $x_{c}(i,j)$ respectively represent the input and output of the *c*-th channel; $g_{c}^{h}(i)$ and $g_{c}^{w}(j)$ represent the attention weights of the *c*-th channel along the *H* and *W* directions.

#### 3.2.2. Bidirectional Feature Pyramid Network

**Figure 7:**
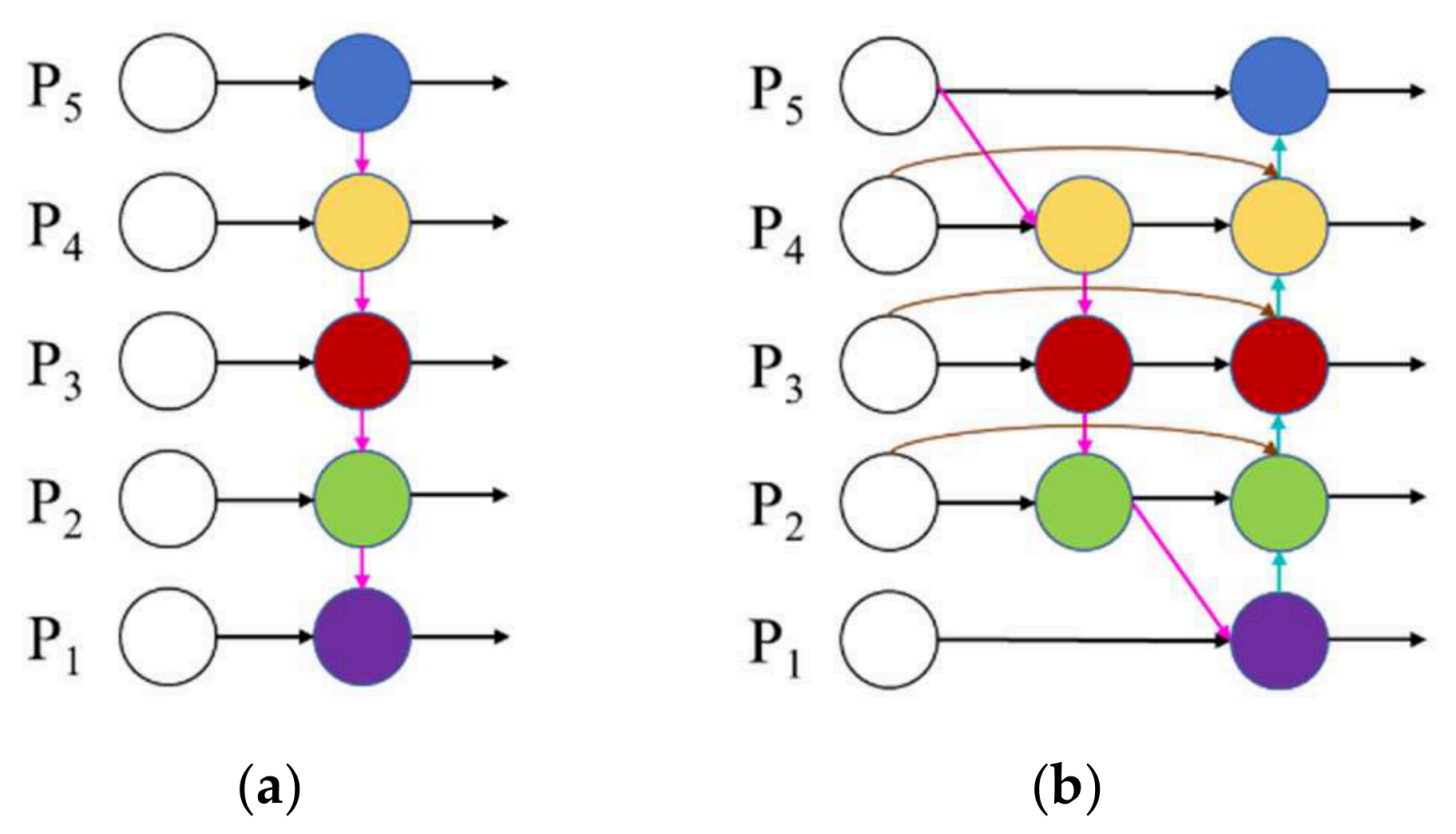
Schematic diagram of FPN series structure. (**a**) FPN. (**b**) BiFPN. Note: P_1_–P_5_ are nodes.

With the reduction in the model parameter quantities and the computation amount, the performance of the model will decrease to a certain extent. It is necessary to adopt strategies to improve the feature extraction ability of the model and ensure its accuracy. In the original YOLOv5s model, a top-down FPN (feature pyramid network) one-way information flow was used. This study adopted an efficient multi-scale feature fusion method BiFPN based on bidirectional cross scale connections and weighted feature fusion, which can extract information for features of different resolutions. While repeatedly fusing multi-scale features from top to bottom and bottom to top, learning weights are introduced to define the importance of different input features. This method can integrate more features without adding too many parameters, providing powerful semantic information for the network and greatly improving the recognition ability of fuzzy and small targets in the image dataset in this study. The schematic diagram of FPN series structure is shown in Figure 7.

BiFPN considers the adjacent top-down and bottom-up paths in each layer of the multi-layer path as a feature network layer. Through additional weighted feature fusion, BiFPN achieves higher accuracy with fewer parameters. The BiFPN layer integrates downsampling features in the backbone and upsampling features in the neck, and has a stronger ability to aggregate features. This article uses BiFPN layer feature fusion to replace concat layer, and the two fusion feature processes formed are shown in Equations (7) and (8):

(7) $$P_{i}^{m}=Conv\left(\frac{\omega_{1}\times P_{i}^{in}+\omega_{2}\times Res\left(P_{i+1}^{in}\right)}{\omega_{1}+\omega_{2}+\varepsilon }\right)$$

(8) $$P_{i}^{out}=Conv\left(\frac{\omega_{1}^{\prime }\times P_{i}^{in}+\omega_{2}^{\prime }\times P_{i}^{m}+\omega_{3}^{\prime }\times Res\left(P_{i-1}^{out}\right)}{\omega_{1}^{\prime }+\omega_{2}^{\prime }+\omega_{3}^{\prime }+\varepsilon }\right)$$

In the formula, $P_{i}^{in}$ is the input feature of layer *I*; $P_{i}^{out}$ is the intermediate feature of level *i* on the top-down path; $P_{i}^{out}$ is output characteristics of layer *i* on the bottom-up path; *ε* is a small value that can reduce numerical instability; *ω* is a learnable weight; *Res* is usually an upsampling or downsampling operation for resolution matching, and *Conv* is a convolutional operation used for feature processing.

#### 3.2.3. Small Object Detection Layer

To ensure that shallow cracked features are not lost and improve the robustness of target recognition for small insect-bitten and overlapping brown rice defects, a small object detection layer has been added to the original YOLOv5s network, as shown in the red dashed box in Figure 5. The specific operation is carried out in layer 19. Firstly, after one convolution, the number of channels in the feature map is changed to 128, and through one upsampling, the feature layer can be weighted and fused with the second layer (BiFPN_Add), replacing tensor concatenation operation of concat. Then, the connection layers of weighted feature fusion layers 21 and 22 are executed to obtain a new small object recognition detection layer with a scale of 160 × 160.

#### 3.2.4. Ablation Experiments

A series of ablation experiments were conducted, starting from the baseline YOLOv5s model and incrementally added the three proposed components: the CA attention mechanism, the BiFPN structure, and the small object detection layer. The results were summarized in Table 2. Overall, each of the three components provided a measurable benefit, and their combination yielded the best overall performance (96.8% mAP@0.5, 96.5% recall).

### 3.3. Model Training and Testing

#### 3.3.1. Environmental Configuration

The experiments were conducted on a workstation equipped with an Intel^®^ Core™ i7-7700 CPU @ 3.6 GHz, 16 GB of RAM, and an NVIDIA GeForce RTX 4060 GPU with 8 GB of VRAM. The operating system was Windows 10, and the development environment was Anaconda with Python 3.10. Considering ease of debugging and code readability, we adopted PyTorch 2.0.1 as the deep learning framework for model construction and improvement. We used standard NMS with a confidence threshold of 0.25 and an IoU threshold of 0.45. No alternative NMS variants were employed; the SGD optimizer with a momentum of 0.937 and weight decay of 0.0005 was used to train 500 epochs, with a batch size of 20 and an initial learning rate of 1 × 10^−5^. After the loss curves converged stably during training, the performance of each algorithm was analyzed.

#### 3.3.2. Setting of Model Training Parameters

**Figure 8:**
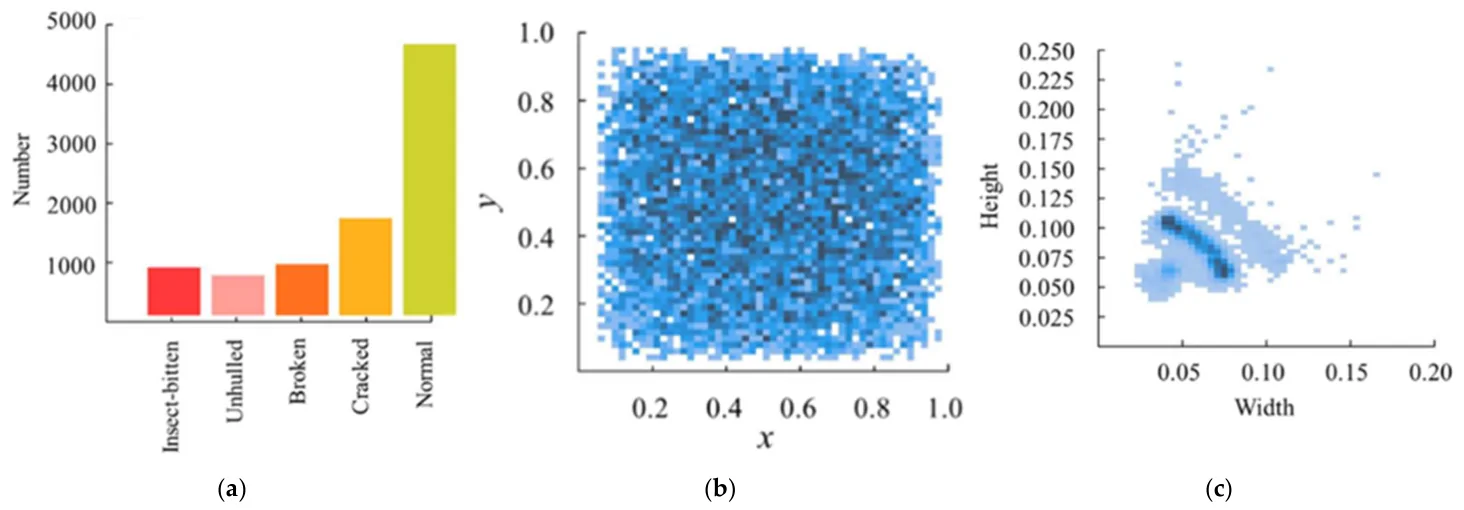
Visual analytics for model training datasets. (**a**) Distribution of number of label. (**b**) Label center coordinate distributions. (**c**) Label width and height distribution.

During model training, the resolution of the training dataset images was adjusted to 640 × 640 pixels. The SGD random gradient descent optimizer was used to train 500 epochs, with a batch size of 20 and an initial learning rate of 10^−5^. The momentum parameter and weight decay parameter were set to 0.937 and 0.0005, respectively. The visualization training results of dataset are shown in Figure 8. Based on the number of labels and object size distribution, it can be seen that there are more positive samples and fewer negative samples, which is in line with the model improvement ideas of this study. Introducing the mosaic data augmentation method during the training process helps to randomly read images from the training set in each epoch for stitching, scaling, translation, rotation, and other operations. Meanwhile, color transformations can be performed on the H (Hue), S (Saturation), and V (Value) color gamut to enhance information expression. This not only improves the number and quality of samples, but also accelerates model convergence.

#### 3.3.3. Evaluating Indicators

After the network model is completed, it is necessary to evaluate the effectiveness of the model and optimize its parameters based on the evaluation results to achieve the expected results. The experimental results use the detection speed FPS (frames per second) as the recognition speed evaluation index of the model, and *P* (Precision, %), *R* (Recall, %), and mAP (mean average precision, %) are used to measure the accuracy of the model prediction [55]. The calculation formulas are as follows:

(9) $$P=\frac{TP}{TP+FP}\times 100\%$$

(10) $$R=\frac{TP}{TP+FN}\times 100\%$$

(11) $$\mathrm{AP}=\int_{0}^{1}P(R)dR$$

(12) $$\mathrm{mAP}(i)=\frac{1}{C}\sum_{1}^{C}AP_{i}(0<i<1)$$

In the formulas, *TP* represents the number of brown rice with surface defects that have been correctly detected; *FP* represents the number of brown rice with surface defects that have been erroneously detected; *FN* represents the number of brown rice undetected in the image; AP is the average value function of *P* for all *R* values between 0 and 1, and *C* is the number of categories to be predicted, with a value of 5 in this article.

## 4. Results and Discussions

### 4.1. Model Performance Comparison

**Figure 9:**
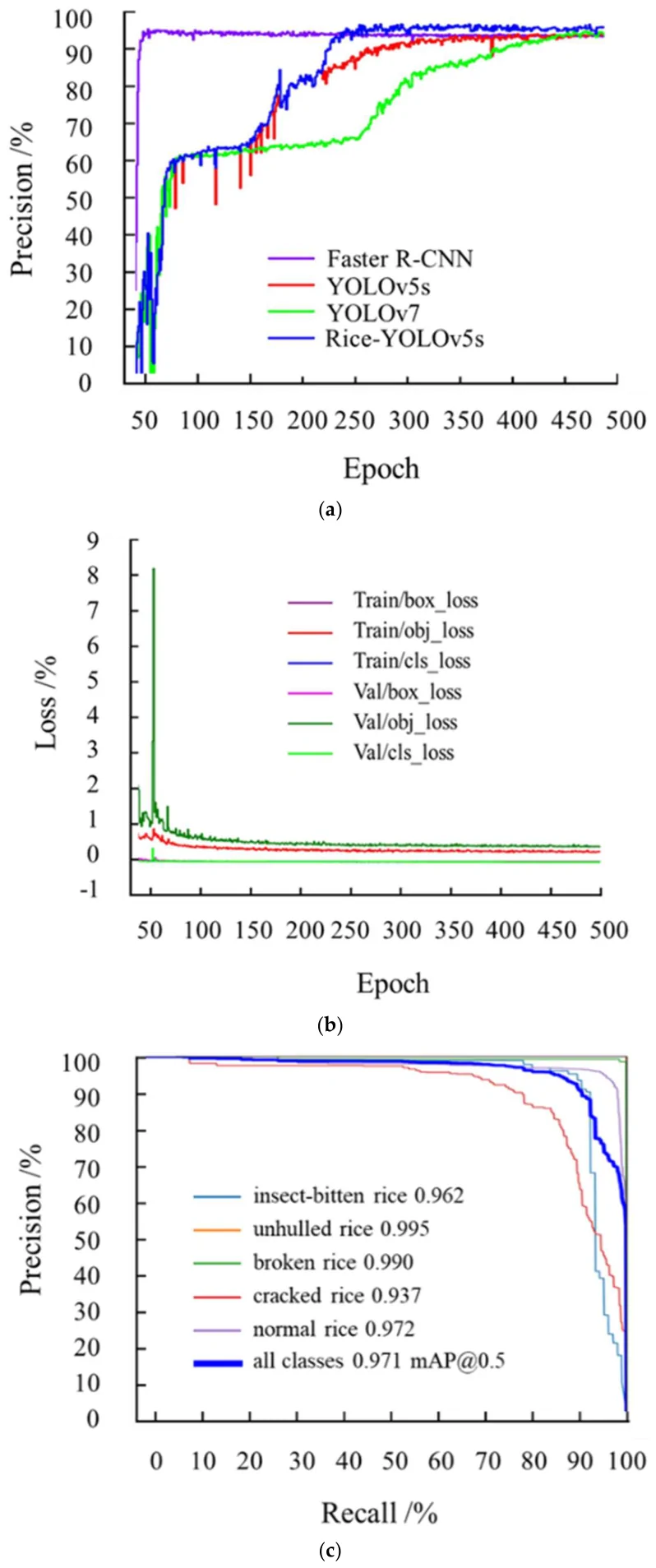
Model training curves of different improved algorithms during training process. (**a**) Precision value change curve of models. (**b**) Rice-YOLOv5s loss value change value. (**c**) Rice-YOLOv5s PR curve.

Another 200 additional images were acquired in a separate, independent acquisition session (December 2025, after the original dataset collection had been completed), which were renamed as independent validation set. These images were taken on different days, under slightly different ambient conditions, and from the same rice variety but from a different batch of grains. They were never used in training or validation at any stage—they served solely as a post-training independent test to evaluate the model’s practical generalization capability under slightly varied conditions. The training results of the model are shown in Figure 9. Figure 9a shows the accuracy performance curves of the four convolutional neural networks as a function of the iterations; it can be seen that Faster R-CNN achieved 95.6% accuracy in the validation dataset after 15 iterations, but then the accuracy slightly decreased and stabilized at around 93.0%. After 500 iterations, the accuracy of the validation dataset for YOLOv5s, YOLOv7, and Rice-YOLOv5s all exceeded 94.3%. YOLOv5s has poor curve dataset generalization ability and is prone to gradient disappearance. The YOLOv7 curve dataset has a small error, but the iteration time for one round is longer. The accuracy of Rice-YOLOv5s reached 96.7%, with an average inference detection time of only 9.2 ms per photo, a detection frame rate of 108.70 FPS, and good generalization ability.

The initialization weights of Rice-YOLOv5s pretreatment training model were used to train the dataset based on hyperparameters. The changes in training loss values and validation loss values are shown in Figure 9b. The smaller the confidence loss, the more accurate the judgment of the target, and the smaller the classification loss, the more accurate the classification. From the graph, it can be seen that after 500 rounds of training, the positioning loss, confidence loss, and classification loss of the loss curve training dataset converge to 0.021, 0.3042, and 0.0095, while the positioning loss, confidence loss, and classification loss of the loss curve validation dataset ultimately converge to 0.017, 0.4375, and 0.010. The loss values in the loss curve training dataset and validation dataset are all low, indicating that the model has good positioning and classification capabilities.

Figure 9c shows the PR curve of the Rice-YOLOv5s model when the intersection and union ratio of the real box and the predicted box is 0.5, as represented by the mAP. From the graph, it can be seen that the improved model has PR curve areas close to 1 for different defect categories of brown rice, indicating that the model has good recognition performance.

**Figure 10:**
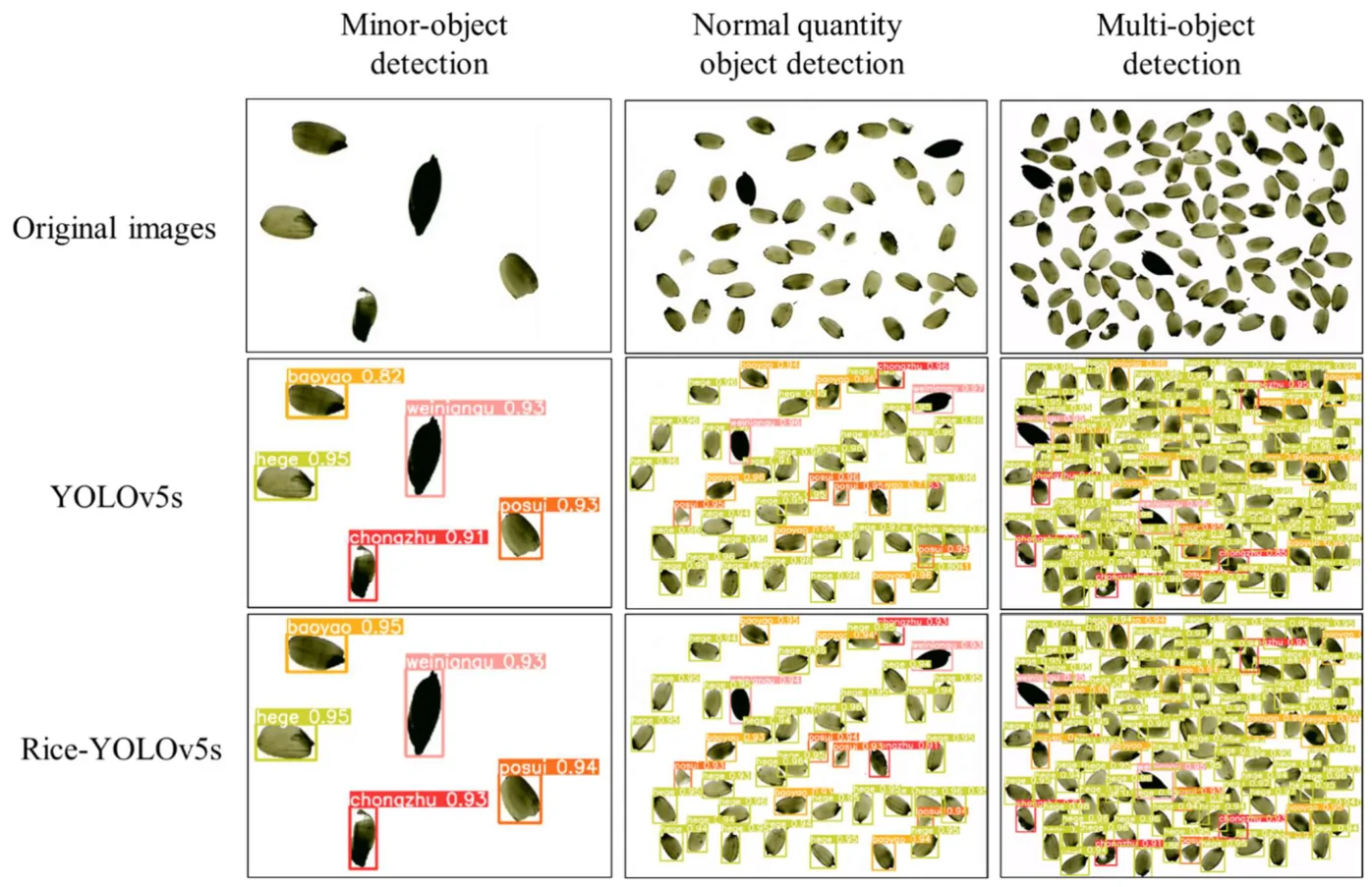
Comparison of detection effect of brown rice full-surface defects before and after improvement of YOLOv5s.

We compared the original YOLOv5s network model with the improved Rice-YOLOv5s model on 108 test sets and randomly selected one brown rice image from three scenarios: minor-object, normal quantity object, and multi-object for detection, as shown in Figure 10. It can be seen that the Rice-YOLOv5s model has a higher confidence performance when detecting brown rice images, and all surface defect targets that appear have been recognized without any missed or multiple detections.

### 4.2. System Validation and Batch Testing Results

**Figure 11:**
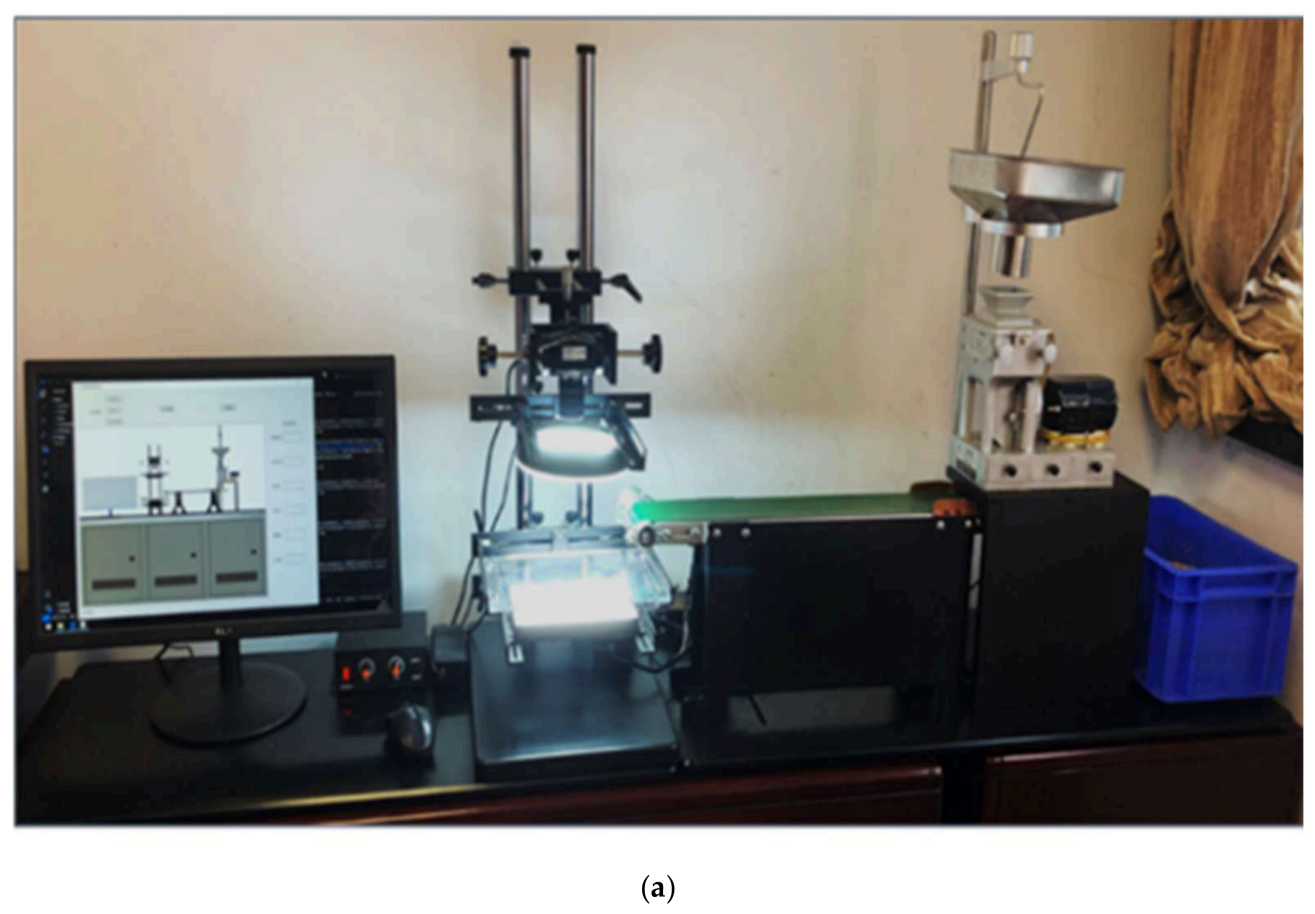

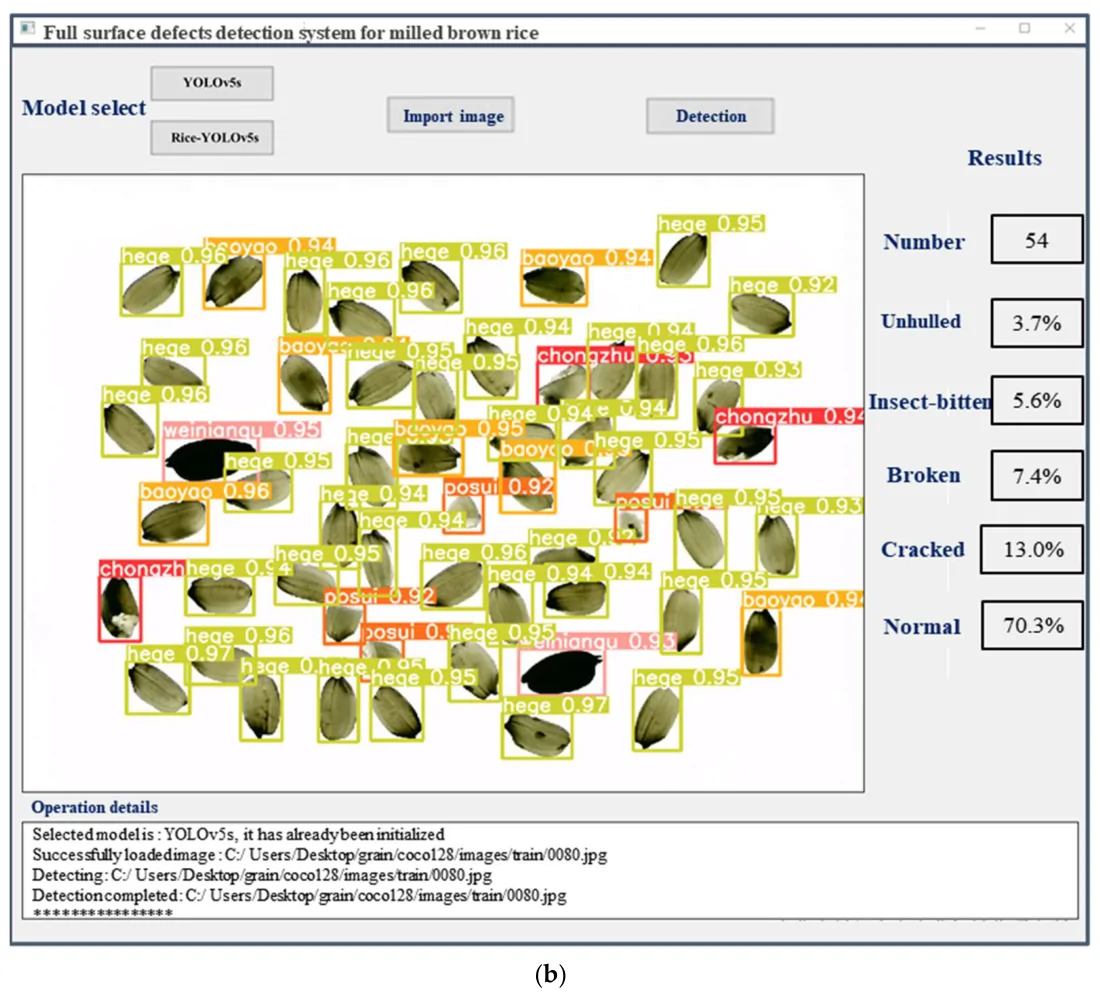
The brown rice full-surface defect recognition system. (**a**) Brown rice full-surface defect recognition system (phototype). (**b**) Recognition software interface.

The brown rice full-surface defect recognition system is shown in Figure 11, which includes the construction of the mechanical device and the installation of the control module. The entire device is covered with black spray paint, which is conducive for the system to identify the transmission of light source to brown rice and prevents other light from interfering with the experiment. The frame itself meets the mechanical requirements and can bear the motor control module and various units. The graphical user interface uses PyQt5, which is a Python library based on the Qt5 framework for creating GUI applications. It provides rich functions and tools to develop cross-platform graphical user interfaces, making it easy to write brown rice full-surface defect recognition software to control each unit device, and output them on the visualization interface in conjunction with recognition algorithms. The interface of the brown rice full-surface defects detection system is shown in Figure 11b, the GUI main file integrates the detection codes of each model, and uses the images collected by industrial cameras. The interface is mainly divided into two parts: the gray setting area and the white view area. In the gray area, some basic button operations are set, such as selecting the detection model, importing images, detecting images, and detecting specific indicators such as total number, unhulled rate, insect-bitten rate, broken rate, cracked rate, qualification rate, etc. The white area is the sampling image after detection, and comparing the images can intuitively reflect the surface defects situation of the sampled brown rice.

For the batch detection experiments, 500 brown rice kernels (100 per category: unhulled, normal, broken, cracked, and insect-damaged) were randomly selected from the independent validation set (200 images) using a random number generator to ensure representativeness; the average detection accuracy for the five types of rice brown rice surface defects was 96.45%. The average detection and recognition time for each group of brown rice was 9.2 ms (Table 3).

The confusion matrix analysis of the experimental results is summarized in Table 4. Based on the confusion matrix, the proposed model demonstrates satisfactory classification performance across all five categories of brown rice samples, with a total of 4822 correctly classified kernels out of 5000, corresponding to an overall accuracy of 96.44%. Among the five categories, unhulled brown rice achieves the highest detection rate of 97.23% with the lowest false negative rate of 2.80%, indicating that this category is least likely to be missed. Normal kernels also exhibit a high precision of 96.92%, suggesting a low false positive rate. In contrast, cracked kernels yield the relatively lowest classification accuracy of 95.73% with the highest false negative rate of 4.30%, implying that this category is the most prone to misclassification. The model achieves over 95% classification accuracy for all categories.

The total number of trainable parameters, computational complexity (FLOPs), and model size for both the baseline YOLOv5s and the improved Rice-YOLOv5s models are summarized in Table 5. The proposed Rice-YOLOv5s achieves a 1.9% improvement in mAP@0.5 (from 95.2% to 97.1%) with only a modest increase in parameters (+0.6 M, +8.6%) and computational cost (+1.7 GFLOPs, +10.3%). This demonstrates that the added modules (CA attention, BiFPN, and small-object detection layer) effectively enhance detection accuracy without introducing excessive computational burden, making the model suitable for real-time deployment in grain processing lines. This finding aligns with the inherent challenges of visual inspection tasks and provides clear guidance for future model improvement.

### 4.3. Discussions and Limitations

This study achieves efficient detection of five types of brown rice surface defects (unhulled, normal, broken, cracked, and insect-bitten) based on an improved YOLOv5s model, with an overall accuracy of 96.44% and an inference speed of 9.2 ms. Compared with the results reported in the recent literature on surface defect detection accuracy for rice quality, our study demonstrates certain advantages in accuracy. For example, the research team at Nanjing Agricultural University conducted a study on internal crack recognition of brown rice based on the YOLOv5s-RF model, achieving an accuracy of 94.01% [56]. The research team at Jiamusi University used the Canny detection model to detect the number of rice grains and cracked brown rice, achieving an accuracy of 88.34% for grain counting and 98% for cracked kernel identification, but the processing time per image reached several seconds [20]. The research team at Heilongjiang Bayi Agricultural University applied a BP neural network combined with near-infrared spectroscopy to identify insect-damaged and moldy rice, achieving an accuracy of 93.33% [10]. Furthermore, in terms of processing speed, our method is superior to or comparable with recently reported image-based detection systems for other grain and oil crops, such as maize and wheat. For instance, the research team at China Agricultural University developed a CNN-based image detection system for internal cracks in maize seeds, with a processing time of 4.42 s per single seed [47]. The research team at Henan University of Technology developed an IDS-YOLO algorithm for detecting imperfect wheat kernels, achieving a detection speed of 88.03 FPS [57]. These comparisons validate the effectiveness of the improved YOLOv5s model under relatively fixed scales and uniform backgrounds.

While the above experimental work has yielded some results, there remain certain aspects that require further improvement. First, in order to further evaluate cross-variety generalization and temporal robustness, future work will expand the dataset to include additional indica and japonica cultivars, multiple harvest years, and different camera setups and lighting conditions, and where feasible, publicly available agricultural image datasets will also be used as external benchmarks. Second, GPU dependency is another area of concern. Although the model is relatively lightweight, its deployment on edge devices still requires GPU acceleration, which may increase hardware costs in practical applications. Third, batch-level data constraints is also an issue; while batch-level splitting effectively prevents data leakage, the overall dataset size remains relatively modest by deep learning standards. These efforts will provide a more comprehensive assessment of the model’s adaptability and practical deployment potential.

## 5. Conclusions

This study demonstrated the feasibility of a CNN-based image recognition system for full-surface defect detection of brown rice. The integration of image analysis, deep learning, and automated decision-making provides a practical solution for intelligent grain quality monitoring, aligning with the development trend of smart analytical technologies for grain inspection. The main conclusions are as follows:

(1) The improved Rice-YOLOv5s model achieves 96.44% detection accuracy and 9.2 ms inference time per image in batch detection experiments, substantially outperforming conventional SVM and BP neural network approaches.
(2) The synergistic combination of CA attention, BiFPN, and a small-object detection layer improves mAP@0.5 from 95.2% to 97.1% with only a modest increase in computational cost (+0.6 M parameters, +1.7 GFLOPs), as validated by ablation experiments.
(3) The complete technical pipeline—from image acquisition and dataset construction to model training and system integration—demonstrates practical engineering value for real-time grain quality inspection.

While the current dataset is limited to a single variety and controlled conditions, and the overall dataset size remains modest by deep learning standards; these limitations have been acknowledged in Section 4.3. Future work will focus on cross-variety data expansion, robust preprocessing for variable imaging environments, and model compression strategies for edge-device deployment. Ultimately, these efforts will push the proposed system toward real-world deployment in rice processing lines.

## Figures and Tables

**Table 1:** **Comparison of object detection algorithms**.

Model	Iteration	Learning Rate	Batchsize	Precision (%)	Recall (%)	Detect Time/ms
BP	500	10^−5^	20	78.2	76.7	4270
SVM	500	10^−5^	20	85.6	84.3	4010
Faster R-CNN	500	10^−5^	20	92.8	91.1	16.72
YOLOv5s	500	10^−5^	20	95.8	94.0	10.87
YOLOv7	500	10^−5^	20	94.3	93.1	14.08

**Table 2:** **Results of ablation experiments**.

Model Configuration	Precision (%)	Recall (%)	mAP@0.5 (%)
YOLOv5s	95.8	94	95.2
YOLOv5s + CA	96.2	94.3	95.7
YOLOv5s + BiFPN	96.1	94.5	95.8
YOLOv5s + small-object layer	95.9	94.2	95.5
YOLOv5s + CA +BiFPN +small-object layer	96.7	96.5	96.8

**Table 3:** **Batch detection accuracy of full-surface defects in brown rice**.

Test No.	Unhulled Rice	Normal Brown Rice	Broken Brown Rice	Cracked Brown Rice	Insect-Bitten Brown Rice	Time (ms)
1	97.35%	97.03%	96.58%	96.08%	95.95%	9.45
2	97.20%	96.92%	97.02%	95.84%	96.29%	8.73
3	97.13%	96.75%	96.05%	95.53%	96.15%	9.91
4	97.46%	96.68%	96.18%	95.66%	95.96%	8.56
5	96.89%	97.11%	96.09%	95.92%	95.87%	9.18
6	97.14%	96.77%	96.43%	95.47%	96.20%	10.03
7	97.16%	96.96%	96.25%	96.20%	95.89%	8.89
8	97.38%	96.83%	96.52%	95.19%	96.27%	9.62
9	97.37%	96.64%	96.14%	95.56%	96.16%	8.34
10	97.25%	96.93%	96.32%	95.89%	95.88%	9.77
Average	97.23%	96.86%	96.36%	95.73%	96.06%	9.20

**Table 4:** **Confusion matrix analysis of the experimental results**.

Actual	Unhulled	Normal	Broken	Cracked	Insect-Bitten	Total
Unhulled	972	9	6	5	8	1000
Normal	11	969	7	7	6	1000
Broken	7	10	964	11	8	1000
Cracked	8	12	14	957	9	1000
Insect-bitten	9	7	10	7	967	1000
Total	1007	1007	1001	987	998	5000

**Table 5:** **Model complexity comparison between baseline YOLOv5s and proposed Rice-YOLOv5s**.

Model	Parameters (M)	FLOPs (G)	Model Size (MB)	mAP@0.5 (%)
YOLOv5s (baseline)	7.0	16.5	14.0	95.2
Rice-YOLOv5s (proposed)	7.6	18.2	15.2	97.1
Increase	0.6	1.7	1.2	1.9

## Data Availability

The original contributions presented in this study are included in the article. Further inquiries can be directed to the corresponding author.

## Abbreviations

*CNN*	Convolutional neural network
*SVM*	Support vector machine
*BP*	Back propagation
*IFA-SVM*	Improved firefly algorithm-support vector machine
*HSI*	Hyperspectral imaging
*ECA*	Efficient channel attention
*SPPF*	Spatial pyramid pooling fast
*FPN*	Feature pyramid networks
*PAN*	Path aggregation networks
*RBF*	Radial basis function
*Conv*	Convolution
*BN*	Batch normalizaion
*CA*	Coordinated attention
*BiFPN*	Bidirectional feature pyramid network
*P*	Precision
*R*	Recall
*mAP*	Mean average precision
